# Trends in Life Expectancy and Lifespan Variation by Educational Attainment: United States, 1990–2010

**DOI:** 10.1007/s13524-015-0453-7

**Published:** 2016-01-26

**Authors:** Isaac Sasson

**Affiliations:** Department of Social Policy, The London School of Economics and Political Science, Houghton Street, London, WC2A 2AE United Kingdom

**Keywords:** Life expectancy, Lifespan variation, Educational disparities in mortality, Race and gender disparities, Vital statistics

## Abstract

The educational gradient in life expectancy is well documented in the United States and in other low-mortality countries. Highly educated Americans, on average, live longer than their low-educated counterparts, who have recently seen declines in adult life expectancy. However, limiting the discussion on lifespan inequality to mean differences alone overlooks other dimensions of inequality and particularly disparities in lifespan variation. The latter represents a unique form of inequality, with higher variation translating into greater uncertainty in the time of death from an individual standpoint, and higher group heterogeneity from a population perspective. Using data from the National Vital Statistics System from 1990 to 2010, this is the first study to document trends in both life expectancy and S_25_—the standard deviation of age at death above 25—by educational attainment. Among low-educated whites, adult life expectancy declined by 3.1 years for women and by 0.6 years for men. At the same time, S_25_ increased by about 1.5 years among high school–educated whites of both genders, becoming an increasingly important component of total lifespan inequality. By contrast, college-educated whites benefited from rising life expectancy and record low variation in age at death, consistent with the shifting mortality scenario. Among blacks, adult life expectancy increased, and S_25_ plateaued or declined in nearly all educational attainment groups, although blacks generally lagged behind whites of the same gender on both measures. Documenting trends in lifespan variation can therefore improve our understanding of lifespan inequality and point to diverging trajectories in adult mortality across socioeconomic strata.

**Electronic supplementary material:**

The online version of this article (doi:10.1007/s13524-015-0453-7) contains supplementary material, which is available to authorized users.

## Introduction

Socioeconomic disparities in health and mortality are perhaps the most fundamental of social inequalities. Educational attainment is a particularly profound predictor of length of life, now surpassing both race (Harper et al. 2007; Kochanek et al. 2013) and gender (Arias 2007; Rogers et al. 2010) in importance in the United States. More troubling is that educational differences in life expectancy have widened dramatically since the 1980s, across all major race and gender groups, and that low-educated white Americans are now seeing absolute declines in longevity (Meara et al. 2008; Montez et al. 2011; Olshansky et al. 2012). Furthermore, those with less than a high school diploma exhibit higher lifespan variability and can expect greater uncertainty in their time of death (Brown et al. 2012; Edwards and Tuljapurkar 2005). By contrast, college-educated Americans live longer, on average, and exhibit greater compression of mortality, with deaths narrowly concentrated at the upper tail of the age distribution—a pattern similarly observed in multiple European countries (van Raalte et al. 2011).

Building on prior research, this study extends the discussion on educational disparities in adult mortality beyond differences in what Cheung and colleagues (2005) term “central longevity indicators”—mean, median, and modal age at death—to include disparities in lifespan variation. The motivation is twofold. First, it facilitates a more nuanced understanding of lifespan inequality and its underlying causes. Higher lifespan variation reflects greater uncertainty in the expected time of death from an individual standpoint (Edwards 2013) and greater heterogeneity in health from a population perspective (Edwards and Tuljapurkar 2005). All else being equal, increasing lifespan variation often corresponds to an increasing proportion of premature and potentially preventable deaths (van Raalte et al. 2011) and should therefore be of interest to both mortality researchers and policy makers. Second, documenting trends in both life expectancy and lifespan variation can provide insight into future mortality scenarios of both advantaged and disadvantaged groups at the subnational level. The scenarios most often considered for the population as a whole are mortality compression (Fries 1980, 1983) and translation (Bongaarts 2005; Bongaarts and Feeney 2003; Canudas-Romo 2008). (I define mortality compression and translation in the upcoming section Future Mortality Scenarios.) Yet, neither of those scenarios has been evaluated for different socioeconomic strata in the United States, particularly over time.

Adopting a multidimensional approach to lifespan inequality, this is the first study to reveal trends in both life expectancy *and* lifespan variation by educational attainment, over a two-decade period, for major race and gender^1^ groups in the United States. The findings replicate and revisit important facts concerning recent trends in life expectancy—namely, that educational disparities are widening among all major gender and race groups and that non-Hispanic white women (but not men) with fewer than 12 years of schooling are increasingly and dramatically worse off in absolute terms. More surprising are trends in lifespan variation. This study finds that between 1990 and 2010, variation in age at death increased among high school–educated Americans in spite of modest gains in life expectancy; at the same time, those with at least some college education have seen tremendous improvements in life expectancy coupled with a steady, record-low variation in age at death. Among women in particular, educational disparities in lifespan variation have become so important that they now approach or even surpass disparities in life expectancy. Overall, these patterns defy the notion that low- and high school–educated Americans are merely lagging behind their college-educated counterparts, as one might conclude from observing trends in life expectancy alone.

Documenting educational differences in the age-at-death distribution requires sample sizes exceeding most survey data, especially when sought in repeated cross-sections. The National Vital Statistics System remains the single most comprehensive source of information on U.S. mortality but suffers from well-known limitations concerning education reporting on death certificates (Rostron et al. 2010). Therefore, before any empirical investigation is pursued, special attention must be given to missing and potentially misreported information on educational attainment in the vital registry. To improve on prior estimates, I develop a unique imputation method to handle missing data in the vital registry, drawing on all available information from observed death records as well as the educational composition of the census (at-risk) population.

In summary, I argue that social disparities in lifespan variation constitute a unique dimension of inequality and, when examined along trends in life expectancy, point to diverging mortality scenarios among educational attainment groups in the United States. This study therefore has three aims:

1. Document change in life expectancy and lifespan variation in the United States from 1990 to 2010 by educational attainment, specific to race and gender subgroups.
2. Examine the relative contribution of differences in means and differences in variances to overall lifespan inequality (i.e., the total divergence between age-at-death distributions).
3. Evaluate whether college-educated Americans exhibit mortality compression or translation, and whether low and high school–educated Americans are following in the former’s footsteps or show diverging trajectories in adult mortality.

## Educational Disparities in Adult Mortality

### Differences in Adult Life Expectancy

One of the most robust and consistent findings in the social sciences is the association between education and adult mortality. Highly educated Americans are subjected to lower mortality rates and consequently have a higher life expectancy than their less-educated counterparts (Hummer and Lariscy 2011). The education-mortality gradient is not only pervasive, spanning multiple race-gender groups and most preventable causes of death (Miech et al. 2011), but also widening over time (Meara et al. 2008; Montez et al. 2011; Preston and Elo 1995) and across successive birth cohorts (Lauderdale 2001; Lynch 2003; Masters et al. 2012).

A recent study further suggested that low-educated, non-Hispanic white Americans—those with fewer than 12 years of schooling—have suffered absolute declines in adult life expectancy in recent decades (Olshansky et al. 2012). Using data from the National Vital Statistics System, that study showed that life expectancy at age 25, *e*_25_^*o*^, declined from 47 to 43.6 years among low-educated white men and from 54.5 to 49.2 years among low-educated white women between 1990 and 2008. By contrast, evidence from the National Health Interview Survey centered on the same period suggests that mortality at ages 45–84 increased among low-educated white women but not among low-educated white men (Montez et al. 2011). Both studies agreed, however, that educational disparities between low- and college-educated whites have widened. The educational gap in *e*_25_^*o*^ increased from 5.1 to 13.2 years among white men and from 1.9 to 10.5 years among white women (Olshansky et al. 2012)—seemingly phenomenal increases in less than two decades. Educational disparities in adult life expectancy also grew among blacks, albeit to a lesser extent, but no absolute decline was observed among blacks with fewer than 12 years of schooling.

Although the widening of educational disparities in adult life expectancy is alarming, several scholars have questioned the causal nature of this association (e.g., Behrman et al. 2011). The expansion of education in American society over the past century rendered the low-educated increasingly (and negatively) select, whereas the college-educated have become less positively select (see Online Resource 1 for change in educational composition by age from 1990 to 2010). The change in educational composition may explain, at least in part, the widening of health disparities (Goesling 2007). Other scholars, however, have emphasized that the risk of mortality diminishes with each additional year of schooling, net of credentials (Montez et al. 2012), and that the protective effect of education grew over time *in spite* of decreasing positive selection among the college-educated (Hayward et al. 2015).

Leaving the causal debate aside, the purpose of this study is to explicate the multiple dimensions of lifespan inequality across educational attainment groups. First, I rely on U.S. vital statistics data to replicate and revisit results reported by Olshansky and colleagues (2012) concerning recent trends in *e*_25_^*o*^ by educational attainment. Using a slightly modified education categorization scheme and a novel method for missing data imputation, I find that both trends—widening educational disparities and also absolute declines in life expectancy among the low-educated—are clearly evident, albeit attenuated compared with those previously reported. In addition to new estimates of *e*_25_^*o*^, this study documents corresponding disparities in lifespan variation and evaluates their importance relative to those in life expectancy.

### Differences in Lifespan Variation

Although group differences in life expectancy constitute the primary and most commonly documented form of lifespan inequality, scholars are increasingly shifting their attention to various measures of lifespan variation (Kannisto 2000) and their characteristics (van Raalte and Caswell 2013; Wilmoth and Horiuchi 1999). Edwards and Tuljapurkar (2005) showed that S_10_, the standard deviation of age at death conditional on survival to age 10, is both informative and distinct from life expectancy as a measure of lifespan inequality. Their study found that around 1981, S_10_ was 2.1 years lower among high school–educated Americans than among those with less than a high school diploma (14.6 and 16.7 years respectively; men and women combined). Although trends in lifespan variation depend on the age *x* on which S_*x*_ is conditioned (Engelman et al. 2010), educational disparities are similarly evident at older ages and in other low-mortality countries. Averaging across 10 European countries in the 1990s, van Raalte and colleagues (2011) found educational differences in S_35_ amounting to 1.5 years among men and 1.4 years among women. They concluded that an excess of premature deaths from circulatory diseases, neoplasms, and external causes was largely responsible for higher lifespan variation among the low-educated.

Emphasizing variation in old-age mortality, other scholars focused instead on the standard deviation around (Canudas-Romo 2008) or above the modal age at death (Cheung and Robine 2007; Thatcher et al. 2010). Still others adopted the Gini coefficient and Theil’s index to examine disparities in the distribution of age at death (van Raalte et al. 2012). Regardless of the measure used, low-education groups consistently exhibit larger variation in age at death compared with their highly educated counterparts (Brown et al. 2012; van Raalte et al. 2012).

In this study, I measure lifespan variation using S_25_ precisely because it captures premature as well as old-age mortality. Conditioning on survival to age 25 ensures that most educational attainment, at least at the college level, is already completed. In addition, I use the Kullback-Leibler divergence (Kullback and Leibler 1951) to evaluate differences across the entire age-at-death distribution and decompose them into contributions from differences in means and differences in variances. A formal definition of the latter follows in the Methodology section.

## Why Variation Matters

### Heterogeneity and Uncertainty

The importance of reducing educational differences in life expectancy is self-evident. But why is tackling other dimensions of lifespan inequality—and, specifically, lifespan variation—so important? Measures of variation play both descriptive and probabilistic roles in population studies (Courgeau 2012). Descriptively, variation measures the spread or dispersion of observations around some central value; probabilistically, variation characterizes a random variable and therefore reflects the uncertainty associated with an individual lifespan. I argue that both interpretations carry relevant information for demographers and policy makers alike.

First, larger variation in age at death implies greater heterogeneity in underlying population health. Highly educated individuals command greater material and nonmaterial resources, which in turn facilitate access to healthier lifestyles and environments (Link and Phelan 1995). In the aggregate, the capacity to optimize health over the life course translates into lower dispersion in the age-at-death distribution relative to subpopulations with fewer resources (Brown et al. 2012). Yet, trends in lifespan variation *within* education groups are equally informative because they summarize changes in underlying age-specific mortality rates. Averting deaths below the young-old threshold age, the age separating early from late deaths (Zhang and Vaupel 2009), *reduces* lifespan variation; averting deaths above the threshold age *increases* variation. In other words, increasing values of S_25_ over time correspond to an increase in premature deaths, a disproportionate decline in old-age mortality, or both (Gillespie et al. 2014). Preventing premature deaths, on the other hand, contributes to significant gains in life expectancy as well as greater equality in individual lifespans (Vaupel et al. 2011).

Second, from a probabilistic standpoint, larger variation corresponds to a higher degree of uncertainty in the individual length of life. Edwards (2013) modeled the economic value of lifespan uncertainty and estimated that the average American would trade roughly one-half year in life expectancy in return for S_10_ that is one year lower. But how do people *really* think and behave? Current evidence suggests that individuals adjust their subjective survival expectations following parental or spousal loss (Hurd and McGarry 2002), that survival expectations vary by socioeconomic status (SES) (Delavande and Rohwedder 2011; Hurd and McGarry 1995) and predict actual mortality (Hurd and McGarry 2002; Perozek 2008), and that individuals base their economic decisions and retirement plans on those expectations (Hurd et al. 2004; van Solinge and Henkens 2010). However, far less is known about the effect of lifespan uncertainty, which is especially onerous among low-educated Americans, on lifelong decision making and the outcomes of those decisions across multiple life domains. This topic not only is ripe for future research but also lies at the heart of population studies, which have long assumed that individuals are aware of demographic reality and change their behavior accordingly (Montgomery 2000). In fact, this exact reasoning underlies classic demographic transition theory, whereby *perceived* improvements in child survival lower the need for parents’ insurance and replacement strategies and therefore drive fertility decline (Lloyd and Ivanov 1988). In this respect, lifespan variation constitutes a fundamental quantity in demographic research.

Although group disparities in lifespan variation reflect inequality at the population level and differential uncertainty at the individual level, long-term trends in variation can also shed light on diverging (or converging) mortality scenarios across educational attainment groups.

### Future Mortality Scenarios

Demographers have long contemplated possible scenarios for the future of human mortality (Wilmoth 2000). Fries (1980) argued that variation in age-at-death will decline as life expectancy approaches the biological limit to human lifespan—a scenario dubbed “the compression of mortality.” Recent evidence suggests, however, that the compression of mortality in high-income countries has slowed down or even stalled since the 1950s, whether measured via S_10_ (Edwards and Tuljapurkar 2005) or the standard deviation about the modal age at death (Canudas-Romo 2008). In light of these findings, scholars have gradually advanced an alternative scenario of mortality translation (i.e., the shifting mortality model) whereby central longevity indicators will continue to increase while lifespan variation remains constant (Bongaarts 2005; Bongaarts and Feeney 2003; Canudas-Romo 2008). In other words, the age-at-death distribution will retain its shape as it shifts to older ages (Ouellette and Bourbeau 2011).

To date, both scenarios have generally been considered for low-mortality countries as a whole. It remains to be seen whether country-level mortality trends are equally shared by subnational socioeconomic strata and, specifically, by various educational attainment groups. Although mortality compression may characterize some education groups, mortality translation might better describe others. Given recent declines in life expectancy among low-educated white Americans, it is clear that certain groups do not partake in either of those favorable scenarios. Yet, several questions remain unanswered. The first relates to the quality of estimates based on U.S. vital statistics. Second, although educational disparities in life expectancy have been studied over time, less is known about trends and disparities in lifespan variation. All evidence from the United States has relied on cross-sectional surveys limited to non-Hispanic whites. Third, temporal trends in lifespan variation can discriminate between competing mortality scenarios among the most socioeconomically advantaged Americans—and suggest new scenarios among less-advantaged groups—that are otherwise not captured by trends in life expectancy alone. Most importantly, evaluating mortality scenarios separately for each educational attainment group will answer whether low-educated and even high school–educated Americans are simply following in the footsteps of the college-educated, albeit at a slower pace, or instead are following alternate and diverging trajectories.

## Methodology

### Data

The basis for all subsequent analyses begins with age-specific mortality rates, with death counts in the numerator and person-years of exposure in the denominator. All-cause death counts were derived from the U.S. Multiple Cause of Death (MCD) public use files (Centers for Disease Control and Prevention 2013) in select census years—1990, 2000, and 2010—and stratified by age, gender, race, and educational attainment. The study period begins in 1990 because educational attainment was not recorded on death certificates nationwide prior to 1989 (National Center for Health Statistics 1993). In the denominator, midyear population estimates were derived from the 5 % Integrated Public Use Microdata Sample (Ruggles et al. 2010) in respective census (and, in 2010, American Community Survey) years. Although education reporting on death certificates suffers from well-known limitations—namely, that educational attainment is reported by someone other than the deceased and therefore heaped at 12 years from both lower and higher levels of education (Rostron et al. 2010; Sorlie and Johnson 1996)—it remains the single most comprehensive data source on U.S. mortality. Furthermore, it is one of the few data sources rich enough to document fine-grained changes in the adult age-at-death distribution by race, gender, and educational attainment.

In both the numerator and denominator data sources, age was recoded to five-year groups starting at 25–29 and ending with an open interval at 90+. Race was categorized as non-Hispanic white and black, excluding other race categories and persons of Hispanic origin because of small death counts or poor reporting (especially in the 1990 MCD). Because the 2000 and 2010 censuses allowed for multiple-race categorization, whereas vital statistics continue to follow single-race categorization, counts from the former were adjusted to match the National Center for Health Statistics’ bridged-race population estimates in respective years (National Center for Health Statistics 2003, 2011). Nationally, race-bridging appears to have only a minor impact on white and black population estimates, adding as much as 0.5 % and 2.5 % to single race counts, respectively, in the 2000 census (Ingram et al. 2003).

In the MCD (numerator) data, educational attainment is classified in single years ranging from zero to five or more years of college. However, in 2003, educational attainment was reclassified on death certificates from single years to completed degrees. Thirty-four states and the District of Columbia adopted the new classification system by 2010, with the remaining 16 states using the old classification (Murphy et al. 2013). To maintain consistency over time, I translated degree categories into completed years of schooling. Most importantly, the new classification collapses “12th grade, no diploma” with the 9–12 years category, leaving high school graduates and general equivalency degree (GED) holders in a separate category. Ignoring the change in classification overestimates the number of deaths among the least-educated while undercounting deaths in the 12 years category. Therefore, for 2010, I reallocated deaths in the 9–12 years group to 0–11 and 12 years proportional to their relative size in the at-risk population by age, gender, and race (shown in Online Resource 1). This approach is likely conservative and accounts for departures from previously published estimates.

Next, I recoded educational attainment in the census (denominator) data to match the MCD classification of completed years of schooling (0–11; 12; 13–15; 16+). All categories below 12 years were recoded as 0–11. Those with more than one year of college education or an associate’s degree were classified as 13–15, and those with a bachelor’s degree or higher were placed in the 16+ category. Finally, all those who reported completing grade 12 (with or without diploma), obtaining a GED, or completing “some college credit, but less than one year” were coded as 12 years. Given that those with some college education but no degree are significantly more likely to be reported as high school graduates on death certificates (Rostron et al. 2010), and probably more so if they had completed less than a full year of college, I included them in the 12 years category (consistent with *completed* years reported in the MCD).^2^ This classification aims to reduce non–sampling error due to education misreporting; it also departs from the categorization used by Olshansky and colleagues (2012) and explains much of the discrepancy between our estimates (see Online Resource 1 for replication of the former). In effect, it serves to inflate the denominator for the 12 years category and hence reduce mortality rates for that group at the expense of the 13–15 category.

### Missing Data Imputation

The MCD suffers from a significant amount of missing data on educational attainment and, to a lesser extent, on Hispanic origin. In 1990, seven states did not report educational attainment on death certificates, and the remaining states had an average of 10.0 % missing information. By 2000, only three states failed to report altogether, and the level of missing data among all other states declined to an average of 4.0 %. Information on state of occurrence is absent from the 2010 MCD public use file; by 2010, however, all states reported (some version of) educational attainment, and missing information declined to 2.3 % nationally. Similarly, three states neglected to report Hispanic origin in 1990, with reporting improving dramatically in 2000 and 2010. Because I exclude Hispanics from all subsequent analyses, the imputation of Hispanic origin serves only to allocate unclassified deaths to non-Hispanic groups. This number amounts to nearly 100,000 deaths in 1990 (most attributed to the three states and New York City failing to report) and is particularly important to include when estimating mortality rates where the numerator and denominator are unlinked.

Imputation of Hispanic origin in the MCD was based on the proportion of non-Hispanics in the census population by gender; race; age; and, where missingness was especially high, by state of occurrence.^3^ Because Hispanics represent a small minority among older U.S. cohorts, imputation is unlikely to jeopardize results for the non-Hispanic majority. On the other hand, estimates for Hispanics would be greatly impacted by underreporting and are therefore not pursued in this study.

Although only the numerator (death counts) had cases with missing educational attainment, the denominator (midyear population estimates) can nonetheless prove useful in imputing the former. Bayes’ rule makes this relationship clear:

1 $$ p\left(\left. Education\right| Death,\mathbf{X}\right)=p\left(\left. Death\right| Education,\mathbf{X}\right)\cdot p\left(\left. Education\right|\mathbf{X}\right)\cdot \frac{1}{p\left(\left. Death\right|\mathbf{X}\right)}, $$

such that the distribution of education in the vital registry, on the left side of Eq. (1), depends on three terms on the right side: (1) the probability of mortality conditional on educational attainment; (2) the marginal distribution of education in the at-risk population; and (3) the marginal distribution of mortality. This relationship can be further conditioned on **X**, a vector of covariates including age group, race, gender, and state of occurrence.

Although Eq. (1) describes a mathematical identity, it can also be used as an imputation device where each component is estimated separately. This approach takes advantage of the fact that for some quantities, population data are practically complete, whereas other quantities involve missing information and must be estimated. Because *p*(*Education*|**X**) depends only on the at-risk population and *p*(*Death*|**X**) does not depend on education, both can be derived from fully observed information.^4^ On the other hand, *p*(*Death*|*Education*, **X**) depends on missing data, as per the original problem, and has to be estimated. I estimate this quantity at the national level using states with nearly complete data—less than 10 % missing—which follows the convention used by official National Center for Health Statistics publications (National Center for Health Statistics 1993).

Using Eq. (1), I could then estimate *p*(*Education* | *Death*, **X**) and the distribution of educational attainment among those who have died (i.e., appear in the vital registry), and imputed missing values by randomly drawing from it conditional on age, race, gender, and state of occurrence.^5^ The last step was repeated 10 times, averaging death counts across iterations to yield the final estimates (see Table 1), although differences between iterations were negligible.

**Table 1:** Death counts by race, gender, and educational attainment: United States, 1990–2010

		Non-Hispanic White		Non-Hispanic Black	
Year	Education	Women	Men	Women	Men
1990	0–11	338,760	342,673	60,615	70,700
	12	338,761	313,921	34,606	41,168
	13–15	97,574	98,480	8,582	10,086
	16+	78,695	116,707	6,847	6,702
	Total	853,790	871,781	110,650	128,656
2000	0–11	314,706	274,279	58,963	57,741
	12	443,496	367,756	49,115	52,230
	13–15	137,133	126,957	14,872	13,589
	16+	106,417	151,847	10,459	9,131
	Total	1,001,752	920,839	133,409	132,691
2010	0–11	203,629	187,152	42,692	42,460
	12	490,761	411,653	55,776	60,979
	13–15	168,322	166,027	22,766	19,857
	16+	124,296	186,733	13,484	11,301
	Total	987,008	951,565	134,718	134,597

This imputation approach is preferable to other methods^6^ because it maximizes the use of available information from both the numerator and denominator. Essentially, it assumes that the relationship between education and mortality is equivalent among observed and unobserved cases, *weighted by the educational composition and overall level of mortality in each age-gender-race subgroup and state of occurrence*—a strategy that is particularly useful in states with a high proportion of missing information.

### Methods

**Fig. 1:**
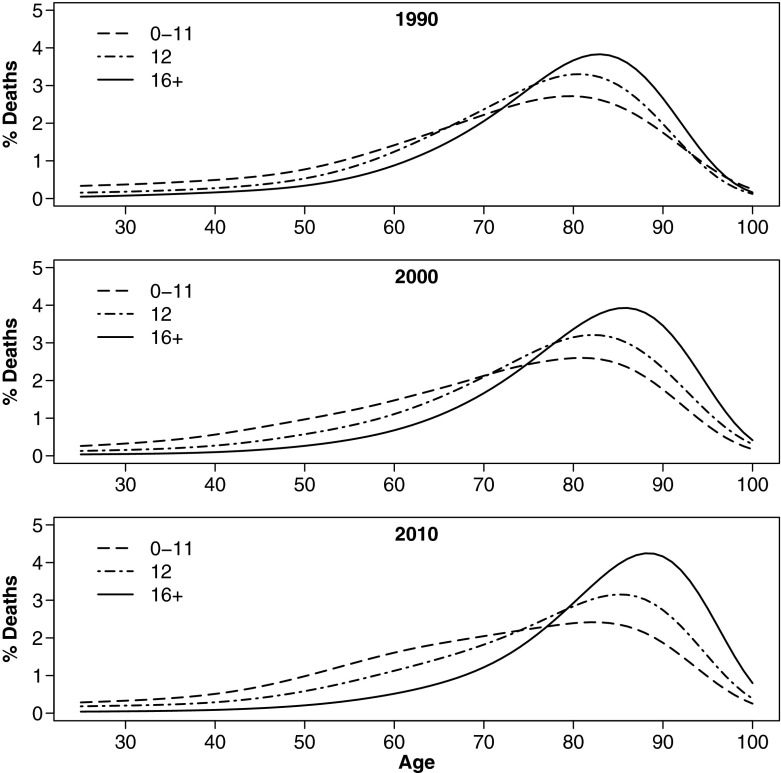
Age-at-death distribution by years of schooling for non-Hispanic white men, 1990–2010

Following missing data imputation, I estimated age-gender-race-education–specific mortality rates as input for standard period life tables. To derive exact age-at-death distributions, I interpolated five-year log-mortality rates to single years using natural cubic splines (Berk 2008) with *n* – 2 knots, where *n* is the number of life-table age groups (excluding the open interval). This approach was used to ensure minimal departure from observed data, as a log-linear (e.g., Gompertz) model might impose, yielding *e*_25_^*o*^ estimates that are comparable with the original five-year life tables. The smoothed age-at-death distributions depicted in Fig. 1 (distributions for all groups are available in Online Resource 1), equivalent to the *d*_*x*_ column in life table notation, were the basis for all subsequent calculations (*e*_25_^*o*^, S_25_, and distributional divergence).

Measuring differences across the entire adult age-at-death distribution is less intuitive than comparing particular moments, such as life expectancy or variance. Perhaps the most common measure of distributional divergence, stemming from information theory (Shannon 1948), is the Kullback-Leibler divergence (KLD) (Kullback and Leibler 1951). It takes the form

2 $$ KLD\left({p}_0,{p}_1\right)={\displaystyle {\int}_{-\infty}^{\infty }{p}_0(x) \log}\left(\frac{p_0(x)}{p_1(x)}\right)dx, $$

where *p*_0_ is the probability function of the reference group, and *p*_1_ is the probability function of the comparison group.^7^ The KLD exhibits several useful properties. First, it is nonnegative, with larger values indicating a higher degree of divergence. Two identical distributions will have a KLD value of 0. Second, if both distributions are normal, the KLD can be decomposed precisely into two additive components (Roberts and Penny 2002) such that3

Term A sums to 0 when the variances are equal, leaving contributions from the difference in means (term B) alone. Similarly, when the means are equal, term B equals 0, and any remaining divergence is attributed to difference in variances (term A). Although age-at-death distributions are generally left-skewed, Edwards and Tuljapurkar (2005) suggested that Eq. (3) remains a useful approximation.

The KLD not only provides insight on educational disparities in mortality across the entire age-at-death distribution, but it can also shed light on the relative importance of each component—difference in means and difference in variances—to overall lifespan inequality. This decomposition is especially revealing when observed over time and can be suggestive of future mortality trends for various social groups as well as disparities between them. However, because the KLD does not indicate the direction of distributional divergence (e.g., which group exhibits lower or higher variance), trends in life expectancy and lifespan variation must be established beforehand.

## Results

### Educational Differences in Life Expectancy

Life expectancy at age 25 by race, gender, and educational attainment is shown in Table 2. Overall, *e*_25_^*o*^ increased by 1.5 years among non-Hispanic white women and by 3.2 years among white men between 1990 and 2010. Gains were higher among non-Hispanic blacks, amounting to 3.2 among women and 6.3 years among men.^8^ Nevertheless, higher gains among blacks reflect a significantly lower starting point relative to whites of the same gender—a gap that was maintained, albeit diminished, over the 20-year period. Consistent with the gender gap in life expectancy, women fare better than men in each race group.

**Table 2:** Life expectancy at age 25 by race, gender, and educational attainment: United States, 1990–2010

	Non-Hispanic White						Non-Hispanic Black					
	Women			Men			Women			Men		
Education (yrs.)	1990	2000	2010	1990	2000	2010	1990	2000	2010	1990	2000	2010
0–11	54.0	51.5	50.9	46.0	45.2	45.4	49.9	49.5	51.8	39.6	42.2	45.5
12	55.1	55.6	55.9	48.7	50.0	50.5	49.2	50.4	52.7	41.4	43.9	46.5
13–15	55.2	56.0	56.7	49.7	52.3	52.8	49.6	51.6	54.2	43.1	48.6	50.9
16+	56.5	58.7	60.2	52.1	54.9	57.3	51.8	54.5	56.5	46.5	50.4	54.1
Total	55.4	55.8	56.9	49.3	51.1	52.5	50.8	51.6	54.0	42.2	45.3	48.5

Several notable patterns arise when broken down by educational attainment. First, educational disparities in life expectancy grew among all race and gender groups from 1990 to 2010. The difference in *e*_25_^*o*^ between the most-educated (16+) and least-educated (0–11) white women grew from 2.5 to 9.3 years; a smaller increase was observed among black women, from 1.9 to 4.7 years. Educational differences in *e*_25_^*o*^ are generally larger among men, and they too experienced widening disparities: from 6.1 to 11.9 years among whites, and from 6.9 to 8.6 years among blacks. Even though educational disparities in mortality are still wider among men, women are slowly catching up.

Second, absolute declines in *e*_25_^*o*^ were observed among the least-educated white men and women, but not among blacks. Within two decades, *e*_25_^*o*^ declined by 3.1 years among white women and by 0.6 years among white men with fewer than 12 years of schooling. At the same time, all other groups experienced gains in life expectancy. Life expectancy among the least-educated black men and women increased by 5.9 and 1.9 years, respectively. Overall, in each race-gender group, the highest gains were experienced by those with 13–15 or 16+ years of education, whereas only modest gains occurred among those with 12 years of education (especially white men and women).

These patterns are generally consistent with those reported by Olshansky and colleagues (2012). However, using the current methodology, the declining trend in life expectancy is significantly attenuated for white women and disappears almost completely for white men. On the whole, the findings suggest that total gains in life expectancy are primarily driven by highly educated Americans, whereas the high school–educated lag behind and the low-educated—at least among whites—are increasingly worse off.

### Educational Differences in Lifespan Variation

Patterns and trends in life expectancy by educational attainment are revealing, but they do not tell the whole story. Additional insights can be gleaned by exploring educational differences in lifespan variation. Trends in the standard deviation of age at death, conditional on survival to age 25, are summarized in Table 3. Overall, race-gender patterns mimic those of life expectancy in reverse. Higher variation is characteristic of men compared with women and blacks compared with whites. Between 1990 and 2010, S_25_ among white women plateaued around 13.2 years and increased only slightly among white men, from 14.1 to 14.5 years. The opposite was recorded among blacks—S_25_ declined from 16.4 to 15.5 years for men and from 15.7 to 14.8 years for women—suggesting that blacks, as a group, are becoming increasingly homogeneous with respect to age at death but are nevertheless disadvantaged compared with whites of the same gender.

**Table 3:** Adult lifespan variation (S_25_) by race, gender, and educational attainment: United States, 1990–2010

	Non-Hispanic White						Non-Hispanic Black					
	Women			Men			Women			Men		
Education	1990	2000	2010	1990	2000	2010	1990	2000	2010	1990	2000	2010
0–11	15.4	16.1	16.8	16.5	16.5	16.7	17.8	17.6	17.2	18.1	17.2	17.3
12	12.9	13.3	14.4	14.1	14.6	15.5	14.8	14.9	15.3	15.4	15.2	15.4
13–15	12.5	12.4	12.9	13.6	13.4	14.0	14.1	14.1	13.9	14.8	15.0	14.7
16+	11.7	11.9	11.3	12.5	12.2	12.2	12.9	14.0	12.5	14.4	14.3	13.3
Total	13.2	13.0	13.3	14.1	13.9	14.5	15.7	15.3	14.8	16.4	15.6	15.5

As with life expectancy, disaggregating trends in S_25_ by educational attainment reveals significant disparities. Across the board, those with higher levels of educational attainment benefit from lower lifespan variation. This finding suggests that the highly educated are not only advantaged with respect to the expected age at death, but also benefit from lower dispersion or less uncertainty around that central indicator. In 2010, differences in S_25_ between the least- and most-educated amounted to more than four years among men and about five years among women, regardless of race. In other words, although educational differences in life expectancy are greater among men, differences in variation are greater among women. Even more telling is the fact that within each race group, low-educated men and women have converged to similar values of S_25_, suggesting that they are now more similar to one another than are their highly educated counterparts.

For some groups, S_25_ has seen little to no change over time. For others, S_25_ changed dramatically over the two-decade period. The least- (0–11) and most-educated (16+) black men each experienced a decline of 0.8 and 1.1 years, respectively, suggesting lower dispersion around *e*_25_^*o*^ (which was also on the rise during this period). On the other hand, several groups experienced a significant increase in variation—almost all of which occurred among the low- and high school–educated. In particular, S_25_ increased by 1.4 to 1.5 years among low-educated white women and among high school–educated whites of both genders. The fact that lifespan variation increased mostly among the high school–educated is disconcerting and counters conventional wisdom based on trends in life expectancy alone (i.e., that the high school–educated merely lag behind the college educated). Only by observing differences and trends in lifespan variation do we notice diverging trajectories for the two groups. Interestingly, aside from black men who were severely disadvantaged at baseline, the college-educated did not exhibit significant declines in S_25_, which is consistent with a shifting mortality scenario rather than further compression. At the same time, the persistence of racial differences in lifespan variation even among the highly educated suggests that further improvements are in fact clearly possible, at least among blacks.

Documenting changes in lifespan variation complements the picture painted by trends in life expectancy. However, an important question remains: what is the relative importance of each component to overall lifespan inequality? Decomposing the Kullback-Leibler divergence provides an approximate answer.

### Convergence and Divergence in Age-at-Death Distributions

The KLD is a unitless quantity and, hence, not as easily interpretable as *e*_25_^*o*^ and S_25_. Nevertheless, it exhibits two important advantages. First, the KLD allows a complete comparison between age-at-death distributions and is not limited to a single dimension of inequality, such as differences in life expectancy. Observed over time, it indicates patterns of convergence or divergence in age-at-death distributions between various subpopulations and can shed light on possible future scenarios. Second, it decomposes (approximately) into two additive terms reflecting the relative importance of differences in means and differences in variances in explaining overall lifespan inequality.

**Fig. 2:**
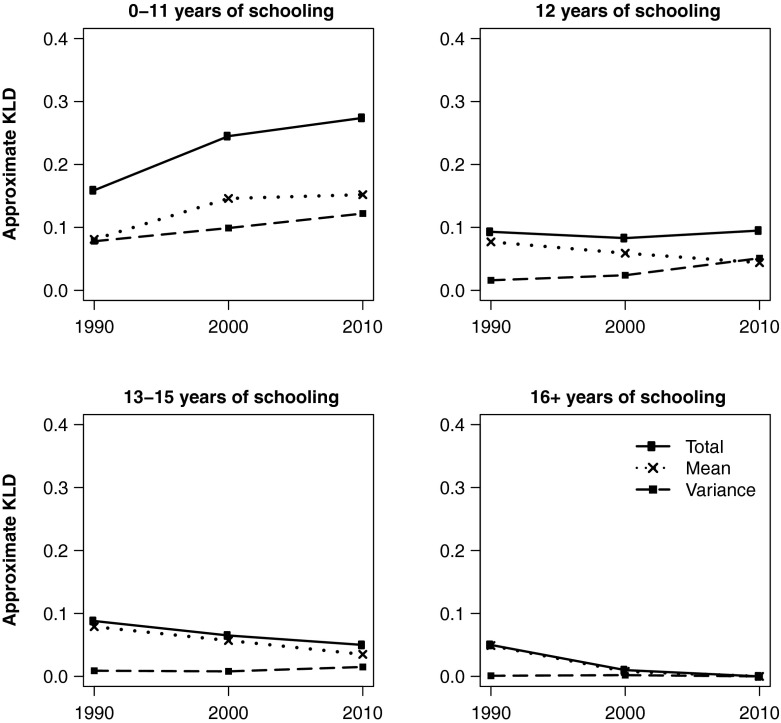
Divergence and convergence in age-at-death distribution by years of schooling for non-Hispanic white women. The reference category is white women with 16+ years of schooling in 2010

**Fig. 3:**
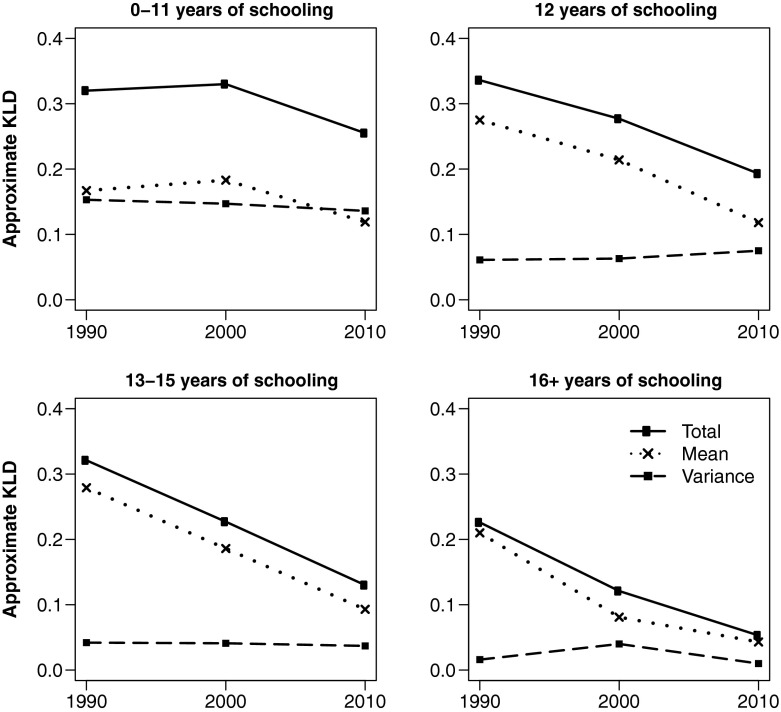
Divergence and convergence in age-at-death distribution by years of schooling for non-Hispanic black women. The reference category is white women with 16+ years of schooling in 2010

**Fig. 4:**
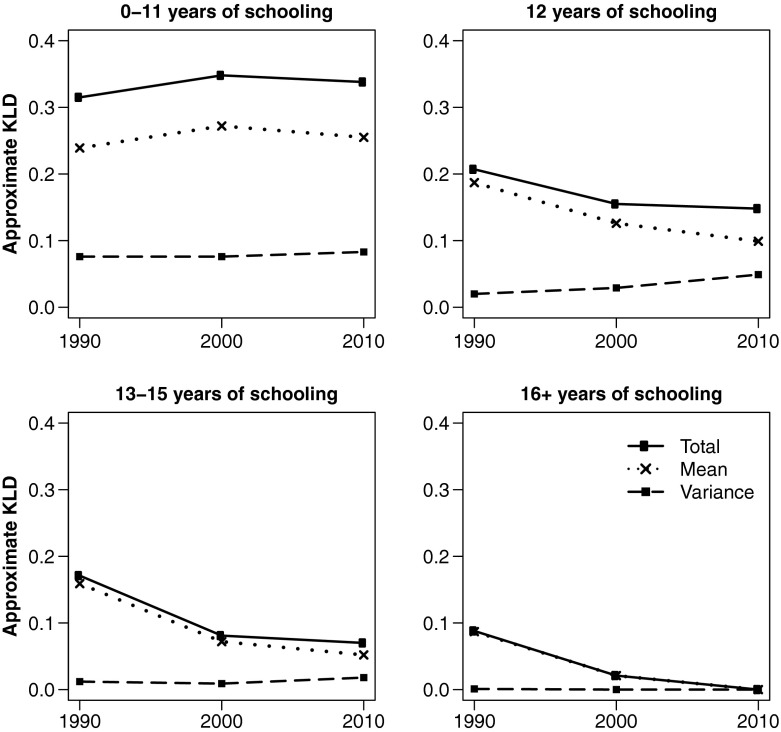
Divergence and convergence in age-at-death distribution by years of schooling for non-Hispanic white men. The reference category is white men with 16+ years of schooling in 2010

**Fig. 5:**
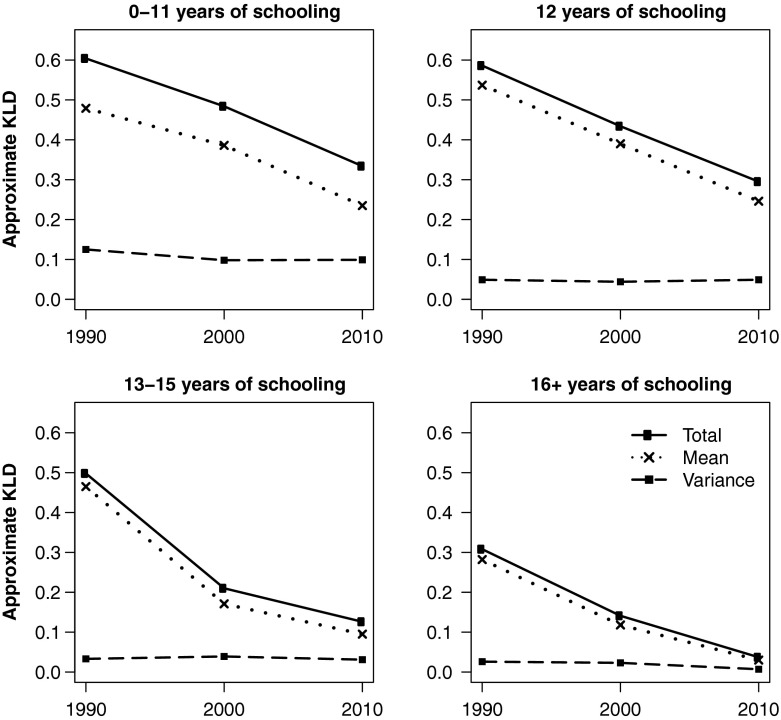
Divergence and convergence in age-at-death distribution by years of schooling for non-Hispanic black men. The reference category is white men with 16+ years of education in 2010. The scale of the vertical axis is different from Figs. 2–4

Figures 2 and 3 depict trends in KLD and its components for white and black women, respectively, by educational attainment. To facilitate comparison across different educational attainment groups, all use the same reference distribution: white women with 16+ years of education in 2010, which is the group with the highest life expectancy and lowest variance in age at death. As of 2010, this group represents the most favorable lifespan conditions for women of either race. Similarly, Figs. 4 and 5 show equivalent trends among white and black men, using white *men* with 16+ years of education in 2010 as reference.

Consistent with the patterns observed in *e*_25_^*o*^ and S_25_, Fig. 2 shows that low-educated white women have continued to diverge from the reference distribution, representing the most favorable gender-specific lifespan conditions, throughout the 1990s and 2000s. During this period, differences in both life expectancy and lifespan variation have contributed significantly to the overall disadvantage, and both components of inequality have been on the rise. Trends among the high school–educated are even more peculiar: whereas differences in life expectancy had declined from 1990 to 2010, differences in variation had increased (especially during the 2000s). In fact, the latter were so pronounced that they now overshadow differences in life expectancy, resulting in net divergence from the reference distribution over time. In other words, lifespan variation is not only increasing in absolute terms, but has now become more important in explaining lifespan disparities between high school– and college-educated white women. Clearly, the shifting mortality scenario does not characterize all educational attainment groups, let alone the low-educated and even high school–educated. Finally, those with some college education appear to follow in the footsteps of the highly educated, which, by design, converge to the reference distribution in 2010.

Figure 3 depicts the results for black women. Similar to whites, lifespan variation contributes more significantly to total divergence among lower education groups—as much as one-half in the case of women with 0–11 years of education. But unlike their white counterparts, all black women converged dramatically toward the reference distribution, primarily because of improvements in life expectancy. Lifespan variation, on the other hand, appears to have plateaued or decreased slightly among all but the high school–educated.

Figures 4 and 5 suggest that lifespan variation plays a smaller role in explaining overall disparities among men compared with women. In Fig. 4, all but the least-educated white men have seen overall improvements in lifespan, with age-at-death distributions converging to that of college-educated men in 2010. Low-educated men, however, diverged from the reference distribution during the 1990s and plateaued during the 2000s. Although differences in means dominated over differences in variation in all educational attainment groups, the latter made a significant contribution to overall lifespan inequality among the low- and high school–educated. Rising lifespan variation among high school–educated men—as is the rise in its relative importance—counters the notion that they merely lag behind their college-educated counterparts, as one might conclude from trends in life expectancy alone. Instead, they are becoming increasingly diverse in their time of death and are (at least in this respect) more dissimilar to the college educated, who experienced continued improvements in life expectancy while lifespan variation remained steady at an all-time low.

Educational disparities in the age-at-death distribution are significantly larger among black men (Fig. 5) than among white men. (Notice the scale change on the vertical axis.) Nevertheless, all educational attainment groups appear to have made progress toward the reference distribution over time because of gains in life expectancy, with some decline in variation as well among the least- and most-educated. As with white men, lifespan variation contributes significantly to overall inequality among the low-educated albeit not as much as it does among women of either race group.

In summary, trends in KLD suggest that almost all but the least-educated white men and women are converging to the age-at-death distribution of their white, college-educated counterparts. At the same time, a troubling pattern emerges. Although high school–educated Americans in all race-gender groups have seen gains in life expectancy, most have also seen increasing heterogeneity in age at death (with the exception of black men, whose initial lifespan variation was highest and remained steady during the two-decade period). Most importantly, lifespan variation is becoming an increasingly important component of lifespan inequality among the high school–educated—often approaching and even surpassing contributions from differences in life expectancy. Focusing on mean-differences alone misses those trends and fosters a false set of suppositions about the future of mortality: namely, that high school–educated Americans, if not the least-educated, are simply lagging behind those with college education. Educational disparities in lifespan variation can therefore point to diverging scenarios in adult mortality.

## Discussion

Educational disparities in U.S. adult mortality have been widening for several decades, with the college-educated faring better than any other educational attainment group (Meara et al. 2008; Montez et al. 2011; Preston and Elo 1995). Most recently, declines in adult life expectancy have been observed among low-educated white Americans (Olshansky et al. 2012)—results that are replicated and updated in the present study. Nevertheless, focusing on life expectancy—a central longevity indicator—overlooks other dimensions of lifespan inequality. Lifespan variation is a particularly revealing dimension of inequality, reflecting within-group heterogeneity in underlying population health (Edwards and Tuljapurkar 2005) as well as the uncertainty associated with an individual lifespan (Edwards 2013). Low-education groups consistently exhibit higher lifespan variation than highly educated groups, regardless of how variation is measured, both in the United States and in Europe (Brown et al. 2012; Edwards and Tuljapurkar 2005; van Raalte et al. 2011). However, no study has yet to document *change* in lifespan variation over time across educational attainment groups. In the United States in particular, previous studies were also limited to non-Hispanic whites or did not disaggregate by race and gender.

Using U.S. vital statistics data from 1990 to 2010, this study reveals trends in adult life expectancy and lifespan variation by educational attainment for black and white men and women. Over the two-decade study period, the gap in life expectancy at age 25 between low- and college-educated whites nearly doubled for men and more than tripled for women, reaching 11.9 and 9.3 years, respectively, by 2010. Among blacks, the same gap amounted to 8.6 for men and 4.7 years for women. Furthermore, *e*_25_^*o*^ declined among low-educated whites by 3.1 years for women and by 0.6 years for men—estimates that are dramatically smaller than those reported by Olshansky and colleagues (2012) but consistent with evidence from the National Health Interview Survey around the same period (Montez et al. 2011). Departures from the former reflect differences in education categorization and a newly developed imputation method to handle missing data in the vital registry.

Disparities in lifespan variation complement those in adult life expectancy and are equally illuminating. Consistent with previous studies, there are large educational differences in S_25_, the standard deviation of age at death conditional on survival to age 25, estimated between 4.0 to 5.5 years across all race-gender groups. From a population perspective, larger variation among lower-education groups reflects the lack of material and nonmaterial resources needed to shape healthier social environments and lifestyles, and therefore a lower capacity to optimize health throughout the life course (Brown et al. 2012). Indeed, since the 1970s, low-educated Americans have faced declining real wages (U.S. Census Bureau 2014), rising poverty and unemployment rates (Pew Research Center 2014), and worsening prospects in the marriage market (Hou and Myles 2008)—all of which are associated with greater risk of mortality (Dupre et al. 2009; Montez and Zajacova 2013; Rogers et al. 2013; Ross and Wu 1995). At the same time, the expansion of education throughout the twentieth century has rendered the low-educated an increasingly select group (Goesling 2007)—possibly on the basis of family social background, which similarly affects adult mortality (Hayward and Gorman 2004). Regardless of the underlying mechanism, these individuals face greater uncertainty regarding their length of life (Edwards 2013), the consequences of which are crucial for understanding social and economic behavior throughout the life course yet remain understudied and undertheorized in population studies.

**Fig. 6:**
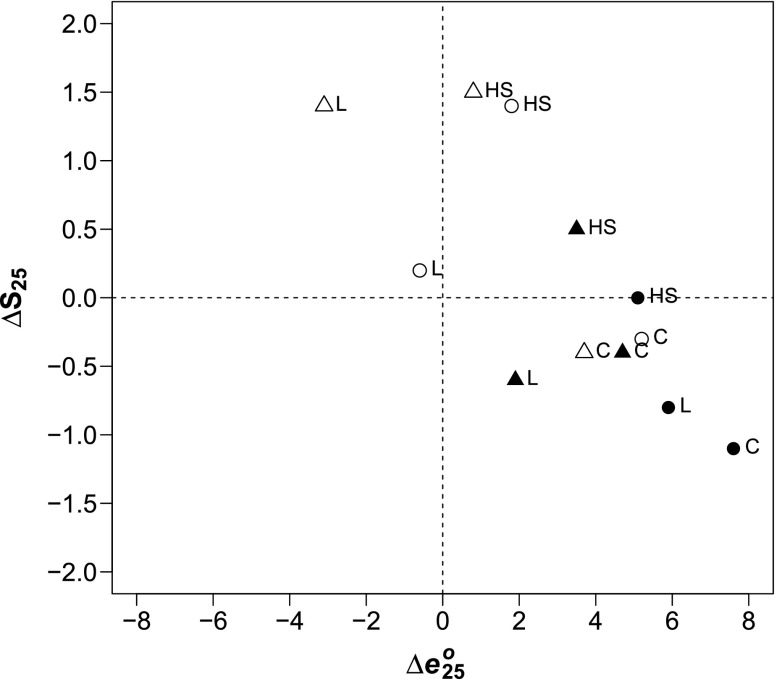
Change in adult life expectancy (*e*_25_^*o*^) and lifespan variation (S_25_) by educational attainment: United States, 1990–2010. Years of schooling: L = 0–11, HS = 12, and C = 16+.  = white women; ○ = white men; ▲ = black women; ● = black men

Over time, changes in lifespan variation provide a glimpse into the future of U.S. mortality and reveal diverging trajectories for various social groups. Researchers have long debated which scenario best describes the future of mortality for low-mortality countries (Canudas-Romo 2008): adult mortality compression, whereby gains in life expectancy are accompanied by decreasing lifespan variation; or shifting mortality, whereby the age-at-death distribution is translated to older ages while retaining its shape. Yet, these scenarios have been considered only for the United States as a whole. In Fig. 6, the change in S_25_ (vertical axis) is plotted against the change in *e*_25_^*o*^ (horizontal axis) for each race-gender-education group, revealing two distinct clusters. The first, in the bottom-right quadrant, consists of college-educated Americans of all race-gender groups and low-educated blacks, for whom *e*_25_^*o*^ increased and S_25_ decreased from 1990 to 2010. Groups in this cluster are generally experiencing mortality translation or, when the vertical component is especially large (e.g., for low- and college-educated black men), mortality compression. The second cluster consists primarily of low-educated and high school–educated whites, for whom S_25_ increased by about 1.5 years, accompanied by losses in *e*_25_^*o*^ (low-educated women) or modest gains at best (high school–educated men and women). This scenario is consistent with increasing young-adult mortality (Gillespie et al. 2014) but warrants additional research as to the underlying change in age-cause–specific mortality rates in those education groups. Across Europe, higher lifespan variation among the low-educated is largely explained by excess young-adult mortality (van Raalte et al. 2011), although less is known about trends over time. The same is likely true in the United States, where young-adult mortality is generally higher than in other high-income countries (Jenkins and Runyan 2005) and educational disparities in premature death rates have recently widened (Ma et al. 2012).

Nevertheless, the findings in this article must be interpreted within the broader context of race, gender, and class inequality in human lifespans. In the United States, gender and race differences in life expectancy have peaked in the late 1970s and early 1990s, respectively, and have since diminished (Arias 2007; Harper et al. 2007; Kochanek et al. 2013). By contrast, educational disparities in life expectancy have increased dramatically over the same period. Borrowing Linton’s (1936) classic typology, it appears that lifespans in the United States are increasingly shaped by achieved, rather than ascribed, status. Although the extent of class mobility in the United States remains debatable (Aaronson and Mazumder 2008; Chetty et al. 2014; Long and Ferrie 2013), the substitution of socioeconomic status for race and gender as the primary determinant of longevity is a recent phenomenon. The same period overlaps with the advent of neoliberal policies (Jacobs and Myers 2014), which have since been adopted around the world (Henisz et al. 2005). Key questions for future research are whether such policies are tied to the changing locus of lifespan inequality and, if so, whether we should anticipate similar trends in other market economies.

This is not to suggest that race and gender no longer matter for individual lifespans. Indeed, they intersect with educational attainment (and, more generally, with socioeconomic status) in several important ways. First, although educational differences in *e*_25_^*o*^ are greater among men, differences in S_25_ are greater among women (for whites and blacks alike). Second, educational differences in both *e*_25_^*o*^ and S_25_ are larger among whites than among blacks, regardless of gender, in spite of the fact that lifespan variability in general is higher among blacks in almost all causes of death (Firebaugh et al. 2014). Third, with respect to race, it appears that education is the overriding factor at the lower end of the distribution—evident in the convergence of *e*_25_^*o*^ and S_25_ between low-educated whites and blacks (in each gender). By contrast, returns to higher education are larger for whites than for blacks, whose lifespans are shorter, on average, and more variable at the college level. Fourth, it appears that college education minimizes the gender gap in *e*_25_^*o*^ but not in S_25_, which is in fact smaller among the low-educated. Perhaps further insight into these new findings can be gained by decomposing differences in lifespan variation by spread, allocation, and timing effects (Nau and Firebaugh 2012).

The results in this study are illuminating, but some limitations should be noted. First, education reporting on death certificates in the National Vital Statistics System is inaccurate (Rostron et al. 2010) and, more generally, estimates derived from census-unlinked data tend to overestimate educational disparities in mortality (Shkolnikov et al. 2007). I attempted to minimize those potential biases by carefully imputing missing data and classifying educational attainment as consistently as possible over time. The assumptions made in the process are conservative yet realistic so as not to inflate disparities—which are in fact smaller than previously reported (Olshansky et al. 2012)—and weigh all available information from the vital registry and census data. Second, the Kullback-Leibler divergence is measured only approximately based on the normality assumption. An exact decomposition into contributions from mean, variance, and residual shape differences to overall lifespan inequality is possible (see Handcock and Morris 1999), although not as straightforward, and can be sought in future studies. Third, trends in life expectancy and lifespan variation are based on period life tables. However, growing educational disparities in mortality during the study period have been driven by cohort effects (Masters et al. 2012), which are also more consistent with the individual experience of disadvantage (and uncertainty) from a life course perspective (Dannefer 2003). Unfortunately, the data requirements for estimating long-term cohort mortality are overwhelming—especially by educational attainment.

In spite of those limitations, a discussion of educational disparities in mortality should not be limited to differences in life expectancy alone. Overlooking disparities in lifespan variation may lead to the erroneous conclusion that high school–educated Americans are merely lagging behind their college-educated counterparts and will eventually catch up. In fact, high school–educated whites (and, to a lesser extent, black women) are becoming increasingly heterogeneous in their lifespans in spite of modest gains in adult life expectancy, a trend suggestive of rising young-adult mortality (Gillespie et al. 2014). Together with low-educated whites, these groups currently account for more than one-quarter of the U.S. population aged 25 and older^9^—hardly a negligible group of “select” individuals—for whom the lifespan is becoming increasingly uncertain. For those on the other end of the distribution, it appears that educational attainment homogenizes not only the life course (Kohli 2007; Oppenheimer et al. 1997; Shanahan 2000) but also the time of death.

## Electronic supplementary material

Below is the link to the electronic supplementary material.

Online Resource 1 (DOCX 756 kb)

## Footnotes

1 Throughout this article, I use the terms “men” and “women” (gender categories) rather than “male” and “female” (sex categories) because differences in longevity between these groups are due to a combination of biological and social factors (Bird and Rieker 2008). However, the U.S. census form and standard death certificate both inquire specifically about sex (which, in the latter case, cannot be self-reported).

2 The 1990 census did not distinguish between those with “some college credit, but less than one year” and “one or more years of college, no degree” as did later years. Hence, in 1990, all those with “some college education but no degree” were reallocated to “12 years” and “13–15” based on the relative proportions by gender and race in the 2000 census.

3 In the state of New York, in 1990, missing Hispanic origins on death certificates were primarily due to nonreporting by New York City and were imputed from metropolitan area, rather than state-level, statistics.

4 Because state of occurrence is not available in the 2010 MCD public use file, *p*(Education | Death, **X**) was estimated directly from observed death records assuming that data are missing at random. In 1990 and 2000, in states with sparse black population, *p*(Death) was estimated using the weighted regression model log(*m*_*ipqr*_) = ∑_*i*_α_*i*_(*State*_*i*_) + *Σ*_*p*_*Σ*_*q*_*Σ*_*r*_β_*pqr*_(*Race*_*p*_ × *Gender*_*q*_ × *Age*_*r*_) + ε_*ipqr*_, where ${\upvarepsilon}_{ipqr}\sim N\left(0,\frac{\upsigma^2}{N_{ipqr}}\right)$, and *N*_*ipqr*_ is the population size in the respective state, race, gender, and age group.

5 The estimated probability mass function is normalized in order to sum to unity.

6 Other methods include imputation based solely on the at-risk population (denominator), solely on deaths records (numerator), or excluding states with incomplete data altogether.

7 Given that the KLD is nonsymmetric—that is, *KLD*(*p*_0_, *p*_1_) ≠ *KLD*(*p*_1_, *p*_0_), in general—the reference distribution, *p*_0_, should be consistent when comparing multiple distributions.

8 Hereafter, non-Hispanic white and non-Hispanic black are referred to as simply white and black, respectively.

9 Author’s calculation of the U.S. Census Integrated Public Use Microdata Sample (Ruggles et al. 2010).
