# Supplementary material for: Trends in Life Expectancy and Lifespan Variation by Educational Attainment: United States, 1990–2010
Source: Demography. 2016 Jan 26;53(2):269–93. doi: 10.1007/s13524-015-0453-7 (PMC4819799; doi:10.1007/s13524-015-0453-7)

Online Resource 1

Trends in Life Expectancy and Lifespan Variation by Educational Attainment: United States, 1990–2010

Isaac Sasson

List of tables:

Table S1: Educational attainment composition by age, non-Hispanic white men 1990-2010

Table S2: Educational attainment composition by age, non-Hispanic white women 1990-2010

Table S3: Educational attainment composition by age, non-Hispanic black men 1990-2010

Table S4: Educational attainment composition by age, non-Hispanic black women 1990-2010

Table S5: Proportion reporting "12th grade, no diploma" among those with 9-12 years of schooling (no diploma), American Community Survey 2010

Table S6: Replication of Olshansky et al. (2012) estimates of e_{25}^{o} for non-Hispanic white men and women, U.S. 1990-2010

List of figures:

Figure S1: Age-at-death distribution by years of schooling, non-Hispanic white men 1990-2010

Figure S2: Age-at-death distribution by years of schooling, non-Hispanic white women 1990-2010

Figure S3: Age-at-death distribution by years of schooling, non-Hispanic black men 1990-2010

Figure S4: Age-at-death distribution by years of schooling, non-Hispanic black women 1990-2010

Table S1: Educational attainment composition by age, non-Hispanic white men 1990-2010

	1990					2000					2010				
Age	0-11	12	13-15	16+	Total	0-11	12	13-15	16+	Total	0-11	12	13-15	16+	Total
25-29	0.10	0.36	0.30	0.25	1.00	0.07	0.38	0.25	0.30	1.00	0.07	0.35	0.27	0.32	1.00
30-34	0.09	0.34	0.30	0.27	1.00	0.07	0.38	0.23	0.32	1.00	0.06	0.35	0.25	0.34	1.00
35-39	0.08	0.29	0.32	0.31	1.00	0.08	0.41	0.22	0.29	1.00	0.06	0.35	0.25	0.35	1.00
40-44	0.09	0.26	0.31	0.35	1.00	0.08	0.41	0.22	0.29	1.00	0.06	0.37	0.24	0.34	1.00
45-49	0.13	0.31	0.26	0.30	1.00	0.07	0.36	0.24	0.33	1.00	0.07	0.40	0.22	0.31	1.00
50-54	0.17	0.34	0.24	0.26	1.00	0.08	0.33	0.23	0.36	1.00	0.07	0.41	0.22	0.30	1.00
55-59	0.22	0.33	0.21	0.24	1.00	0.12	0.38	0.19	0.31	1.00	0.06	0.36	0.24	0.34	1.00
60-64	0.28	0.32	0.19	0.21	1.00	0.15	0.40	0.18	0.27	1.00	0.07	0.33	0.23	0.37	1.00
65-69	0.30	0.33	0.18	0.18	1.00	0.20	0.39	0.15	0.25	1.00	0.10	0.37	0.20	0.33	1.00
70-74	0.36	0.33	0.17	0.15	1.00	0.24	0.39	0.14	0.23	1.00	0.13	0.40	0.18	0.29	1.00
75-79	0.44	0.29	0.14	0.13	1.00	0.27	0.39	0.14	0.21	1.00	0.16	0.40	0.16	0.29	1.00
80-84	0.50	0.24	0.13	0.13	1.00	0.30	0.38	0.13	0.18	1.00	0.20	0.39	0.14	0.27	1.00
85-89	0.57	0.20	0.11	0.12	1.00	0.35	0.37	0.12	0.17	1.00	0.20	0.39	0.14	0.27	1.00
90+	0.60	0.19	0.10	0.10	1.00	0.43	0.30	0.10	0.17	1.00	0.24	0.40	0.13	0.24	1.00

Source: Author's calculation of the U.S. Census Integrated Public Use Microdata Sample and American Community Survey (Ruggles et al. 2010).

Table S2: Educational attainment composition by age, non-Hispanic white women 1990-2010

	1990					2000					2010				
Age	0-11	12	13-15	16+	Total	0-11	12	13-15	16+	Total	0-11	12	13-15	16+	Total
25-29	0.08	0.33	0.33	0.25	1.00	0.06	0.33	0.27	0.35	1.00	0.05	0.26	0.28	0.42	1.00
30-34	0.07	0.34	0.34	0.25	1.00	0.06	0.36	0.25	0.34	1.00	0.05	0.26	0.27	0.42	1.00
35-39	0.07	0.33	0.33	0.27	1.00	0.06	0.39	0.26	0.30	1.00	0.04	0.28	0.28	0.40	1.00
40-44	0.08	0.35	0.31	0.26	1.00	0.06	0.40	0.25	0.29	1.00	0.05	0.32	0.26	0.37	1.00
45-49	0.12	0.39	0.28	0.21	1.00	0.06	0.39	0.25	0.31	1.00	0.05	0.37	0.27	0.32	1.00
50-54	0.16	0.43	0.24	0.16	1.00	0.07	0.42	0.23	0.28	1.00	0.05	0.39	0.26	0.30	1.00
55-59	0.21	0.45	0.21	0.13	1.00	0.11	0.47	0.20	0.23	1.00	0.05	0.39	0.25	0.31	1.00
60-64	0.25	0.44	0.19	0.12	1.00	0.15	0.50	0.17	0.18	1.00	0.07	0.42	0.23	0.29	1.00
65-69	0.28	0.44	0.18	0.10	1.00	0.19	0.51	0.15	0.15	1.00	0.10	0.47	0.20	0.23	1.00
70-74	0.35	0.41	0.15	0.09	1.00	0.22	0.51	0.14	0.13	1.00	0.13	0.50	0.18	0.19	1.00
75-79	0.43	0.35	0.14	0.09	1.00	0.26	0.50	0.13	0.11	1.00	0.17	0.52	0.16	0.16	1.00
80-84	0.47	0.30	0.14	0.10	1.00	0.31	0.46	0.12	0.11	1.00	0.19	0.52	0.15	0.14	1.00
85-89	0.52	0.26	0.14	0.08	1.00	0.36	0.41	0.11	0.12	1.00	0.21	0.53	0.14	0.12	1.00
90+	0.56	0.25	0.12	0.07	1.00	0.41	0.36	0.11	0.13	1.00	0.27	0.48	0.13	0.13	1.00

Source: Author’s calculation of the U.S. Census Integrated Public Use Microdata Sample and American Community Survey (Ruggles et al. 2010).

Table S3: Educational attainment composition by age, non-Hispanic black men 1990-2010

	1990					2000					2010				
Age	0-11	12	13-15	16+	Total	0-11	12	13-15	16+	Total	0-11	12	13-15	16+	Total
25-29	0.18	0.44	0.29	0.10	1.00	0.15	0.50	0.23	0.13	1.00	0.15	0.43	0.28	0.14	1.00
30-34	0.18	0.41	0.29	0.12	1.00	0.14	0.51	0.22	0.13	1.00	0.14	0.44	0.27	0.16	1.00
35-39	0.18	0.38	0.30	0.14	1.00	0.15	0.50	0.23	0.13	1.00	0.11	0.44	0.28	0.17	1.00
40-44	0.21	0.36	0.28	0.15	1.00	0.16	0.47	0.23	0.14	1.00	0.11	0.46	0.26	0.17	1.00
45-49	0.29	0.36	0.22	0.13	1.00	0.16	0.45	0.23	0.16	1.00	0.14	0.47	0.24	0.16	1.00
50-54	0.36	0.35	0.19	0.11	1.00	0.19	0.43	0.21	0.16	1.00	0.15	0.45	0.24	0.16	1.00
55-59	0.46	0.29	0.15	0.10	1.00	0.27	0.42	0.17	0.14	1.00	0.16	0.43	0.25	0.17	1.00
60-64	0.55	0.26	0.12	0.08	1.00	0.36	0.39	0.15	0.11	1.00	0.20	0.41	0.23	0.17	1.00
65-69	0.61	0.24	0.10	0.05	1.00	0.45	0.34	0.12	0.10	1.00	0.26	0.41	0.17	0.16	1.00
70-74	0.69	0.20	0.07	0.04	1.00	0.50	0.32	0.09	0.09	1.00	0.32	0.38	0.16	0.13	1.00
75-79	0.74	0.16	0.05	0.04	1.00	0.57	0.27	0.08	0.08	1.00	0.39	0.36	0.14	0.11	1.00
80-84	0.80	0.12	0.05	0.03	1.00	0.62	0.24	0.07	0.07	1.00	0.46	0.31	0.11	0.12	1.00
85-89	0.82	0.11	0.04	0.03	1.00	0.67	0.22	0.06	0.06	1.00	0.55	0.29	0.09	0.08	1.00
90+	0.84	0.09	0.04	0.03	1.00	0.71	0.20	0.05	0.05	1.00	0.60	0.22	0.08	0.11	1.00

Source: Author’s calculation of the U.S. Census Integrated Public Use Microdata Sample and American Community Survey (Ruggles et al. 2010).

Table S4: Educational attainment composition by age, non-Hispanic black women 1990-2010

	1990					2000					2010				
Age	0-11	12	13-15	16+	Total	0-11	12	13-15	16+	Total	0-11	12	13-15	16+	Total
25-29	0.14	0.39	0.35	0.13	1.00	0.11	0.44	0.28	0.18	1.00	0.10	0.34	0.34	0.22	1.00
30-34	0.15	0.37	0.34	0.14	1.00	0.10	0.45	0.28	0.17	1.00	0.09	0.34	0.33	0.23	1.00
35-39	0.15	0.37	0.32	0.16	1.00	0.11	0.45	0.28	0.16	1.00	0.08	0.34	0.34	0.24	1.00
40-44	0.18	0.38	0.29	0.15	1.00	0.12	0.44	0.27	0.17	1.00	0.09	0.39	0.31	0.22	1.00
45-49	0.25	0.38	0.24	0.13	1.00	0.13	0.44	0.25	0.18	1.00	0.10	0.39	0.30	0.20	1.00
50-54	0.33	0.37	0.19	0.11	1.00	0.17	0.45	0.22	0.17	1.00	0.11	0.39	0.30	0.20	1.00
55-59	0.42	0.33	0.15	0.10	1.00	0.24	0.45	0.18	0.14	1.00	0.13	0.40	0.27	0.20	1.00
60-64	0.50	0.31	0.12	0.08	1.00	0.32	0.43	0.14	0.11	1.00	0.17	0.41	0.24	0.19	1.00
65-69	0.58	0.26	0.09	0.06	1.00	0.40	0.38	0.11	0.11	1.00	0.23	0.43	0.20	0.14	1.00
70-74	0.64	0.23	0.07	0.06	1.00	0.47	0.35	0.09	0.09	1.00	0.31	0.41	0.16	0.12	1.00
75-79	0.71	0.19	0.05	0.05	1.00	0.54	0.31	0.07	0.08	1.00	0.36	0.39	0.13	0.11	1.00
80-84	0.73	0.17	0.05	0.05	1.00	0.60	0.27	0.07	0.07	1.00	0.44	0.35	0.12	0.10	1.00
85-89	0.78	0.14	0.05	0.04	1.00	0.64	0.24	0.05	0.07	1.00	0.48	0.36	0.10	0.07	1.00
90+	0.80	0.12	0.05	0.03	1.00	0.68	0.20	0.05	0.06	1.00	0.54	0.30	0.07	0.09	1.00

Source: Author’s calculation of the U.S. Census Integrated Public Use Microdata Sample and American Community Survey (Ruggles et al. 2010).

Table S5: Proportion reporting "12th grade, no diploma" among those with 9-12 years of schooling (no diploma), American Community Survey 2010

	Non-Hispanic White		Non-Hispanic Black	
Age	Men	Women	Men	Women
25-29	0.215	0.194	0.192	0.195
30-34	0.224	0.171	0.189	0.200
35-39	0.211	0.185	0.193	0.205
40-44	0.255	0.207	0.216	0.219
45-49	0.218	0.216	0.212	0.221
50-54	0.240	0.210	0.222	0.235
55-59	0.247	0.236	0.235	0.207
60-64	0.240	0.225	0.207	0.189
65-69	0.231	0.220	0.197	0.189
70-74	0.245	0.220	0.185	0.166
75-79	0.236	0.219	0.216	0.197
80-84	0.231	0.221	0.194	0.184
85-89	0.241	0.223	0.192	0.175
90+	0.286	0.221	0.233	0.180

Table S6: Replication of Olshansky et al. (2012) estimates of e_{25}^{o} for non-Hispanic white men and women, U.S. 1990-2010

Years of schooling	Olshansky et al. 2012				Sasson (replication)			
	Men		Women		Men		Women	
	1990	2000	1990	2000	1990	2000	1990	2000
0-11	47.0	45.3	54.5	51.4	46.7	45.4	54.3	51.6
12	46.5	47.9	53.6	53.8	47.1	47.9	54.0	53.8
13-15	53.5	55.0	58.2	58.9	52.8	55.1	57.9	59.0
16+	52.1	54.7	56.4	58.4	51.6	54.5	56.3	58.4
Total	49.3	51.0	55.4	55.6	49.3	50.9	55.4	55.7

* Results are based on data from the National Vital Statistics System and the Census Integrated Public Use Microdata Samples for 1990 and 2000. In order to replicate Olshansky et al. (2012) as closely as possible, the same coding scheme was adopted for educational attainment categories; because these authors did not report how they handled missing data on educational attainment, the latter were imputed by assuming equal risk of mortality across educational attainment groups subject to their relative proportion in the census population. The similarity between the replication and Olshansky et al. (2012) suggests that any remaining discrepancies between the latter and the results reported in the main text (e.g., Table 2) are driven by different educational attainment categorization and, to a lesser extent, the Bayesian imputation method (Eq. 1).

Figure S1: Age-at-death distribution by years of schooling, non-Hispanic white men 1990-2010

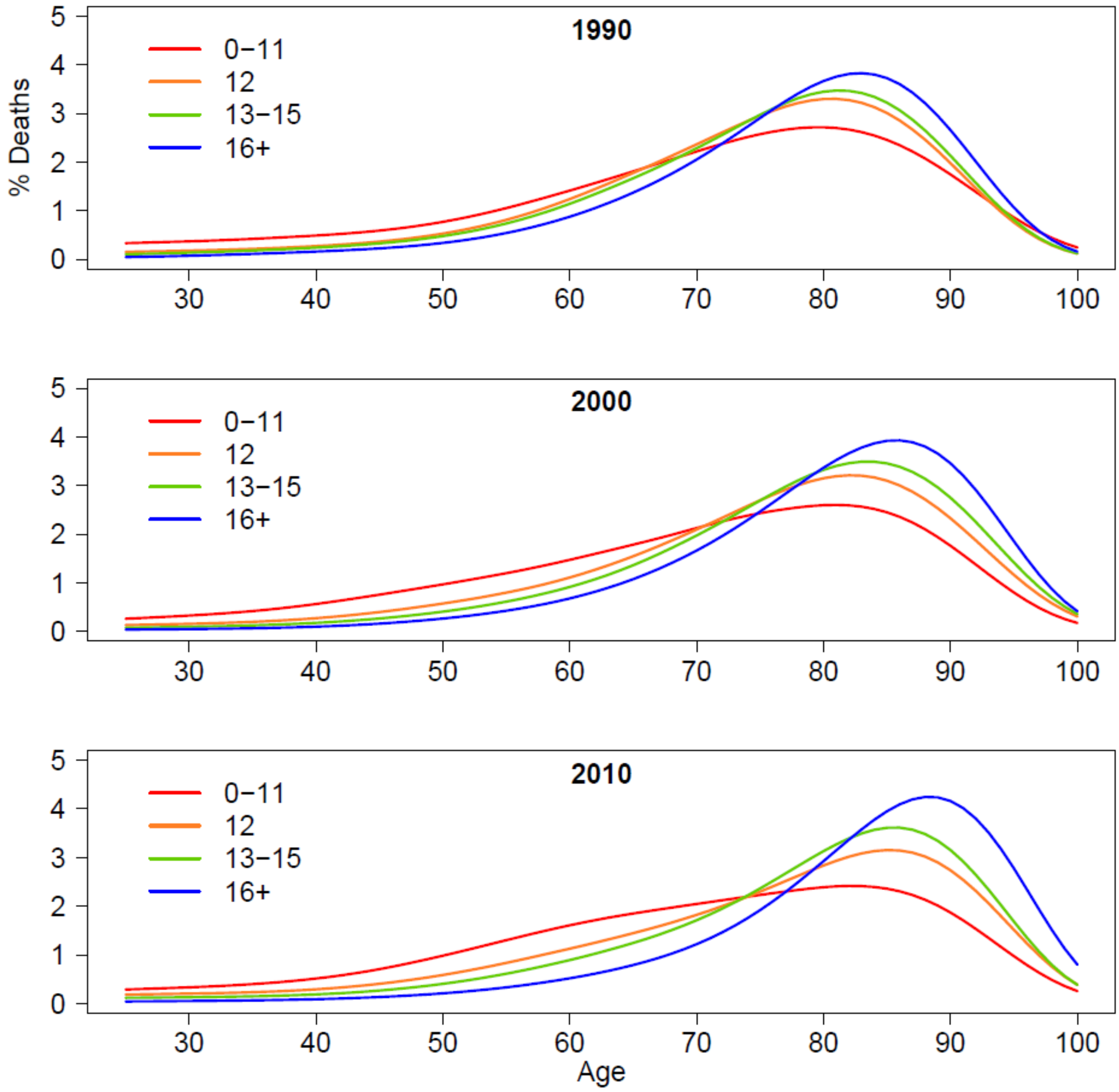

Figure S2: Age-at-death distribution by years of schooling, non-Hispanic white women 1990-2010

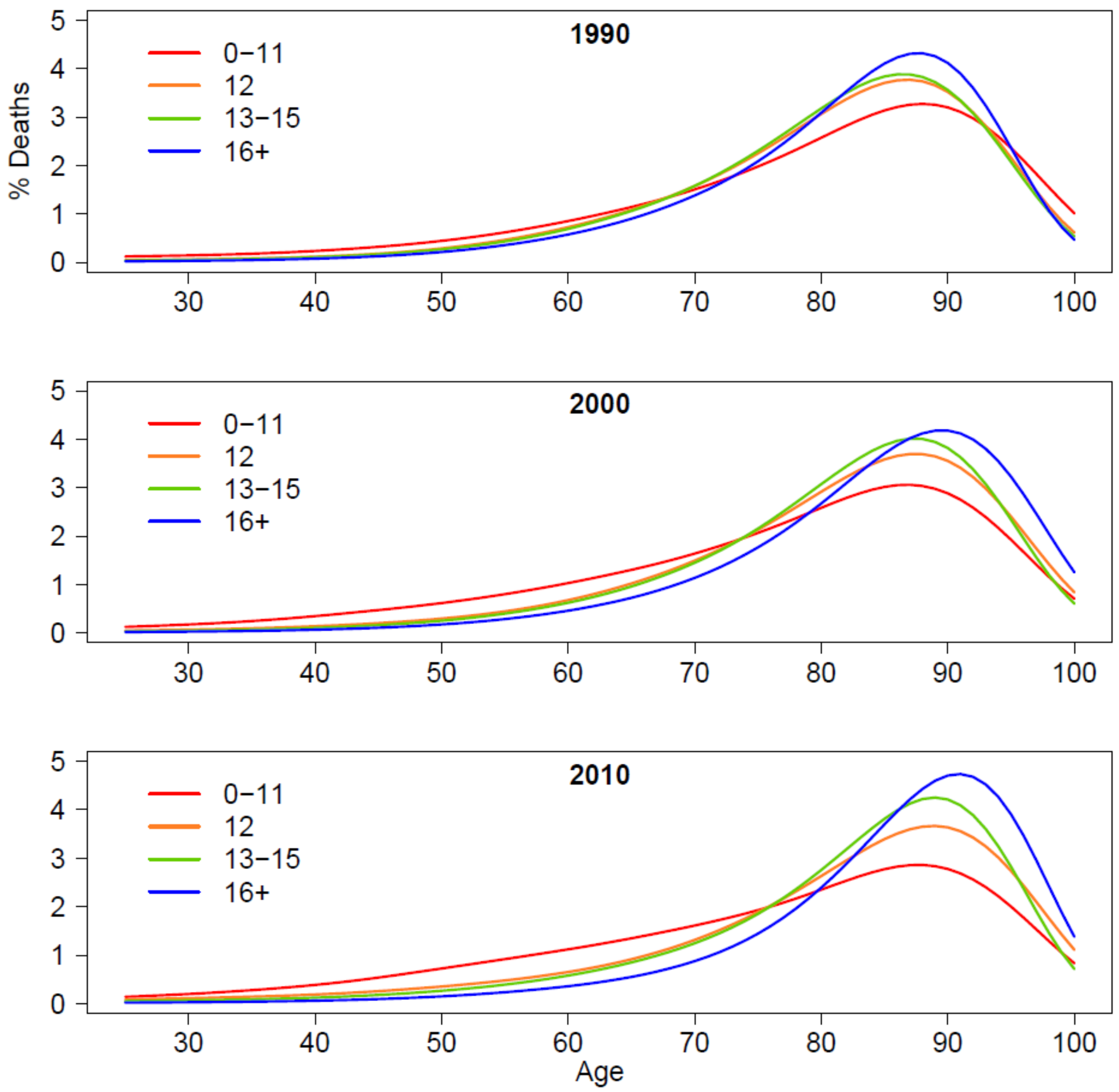

Figure S3: Age-at-death distribution by years of schooling, non-Hispanic black men 1990-2010

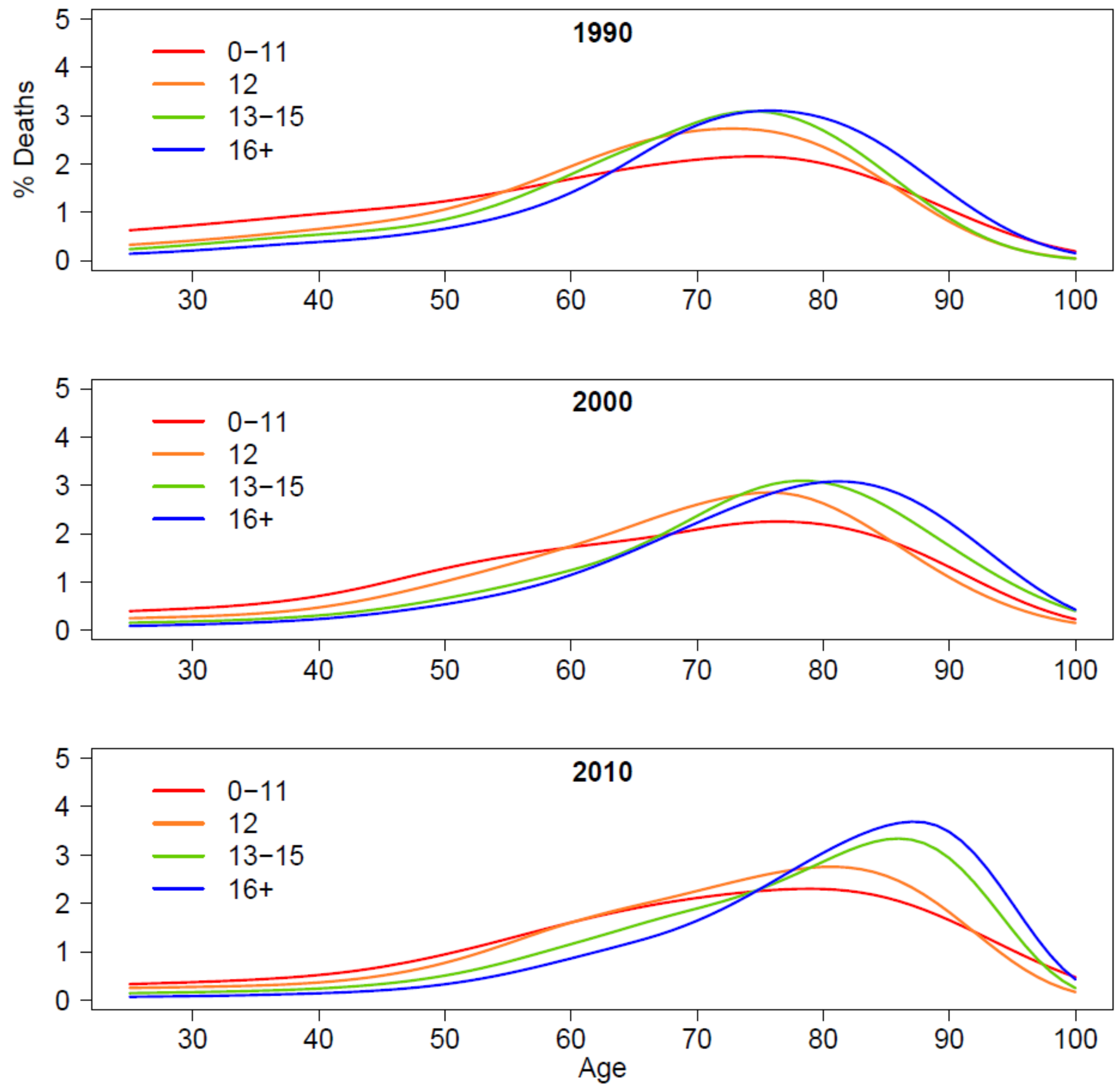

Figure S4: Age-at-death distribution by years of schooling, non-Hispanic black women 1990-2010

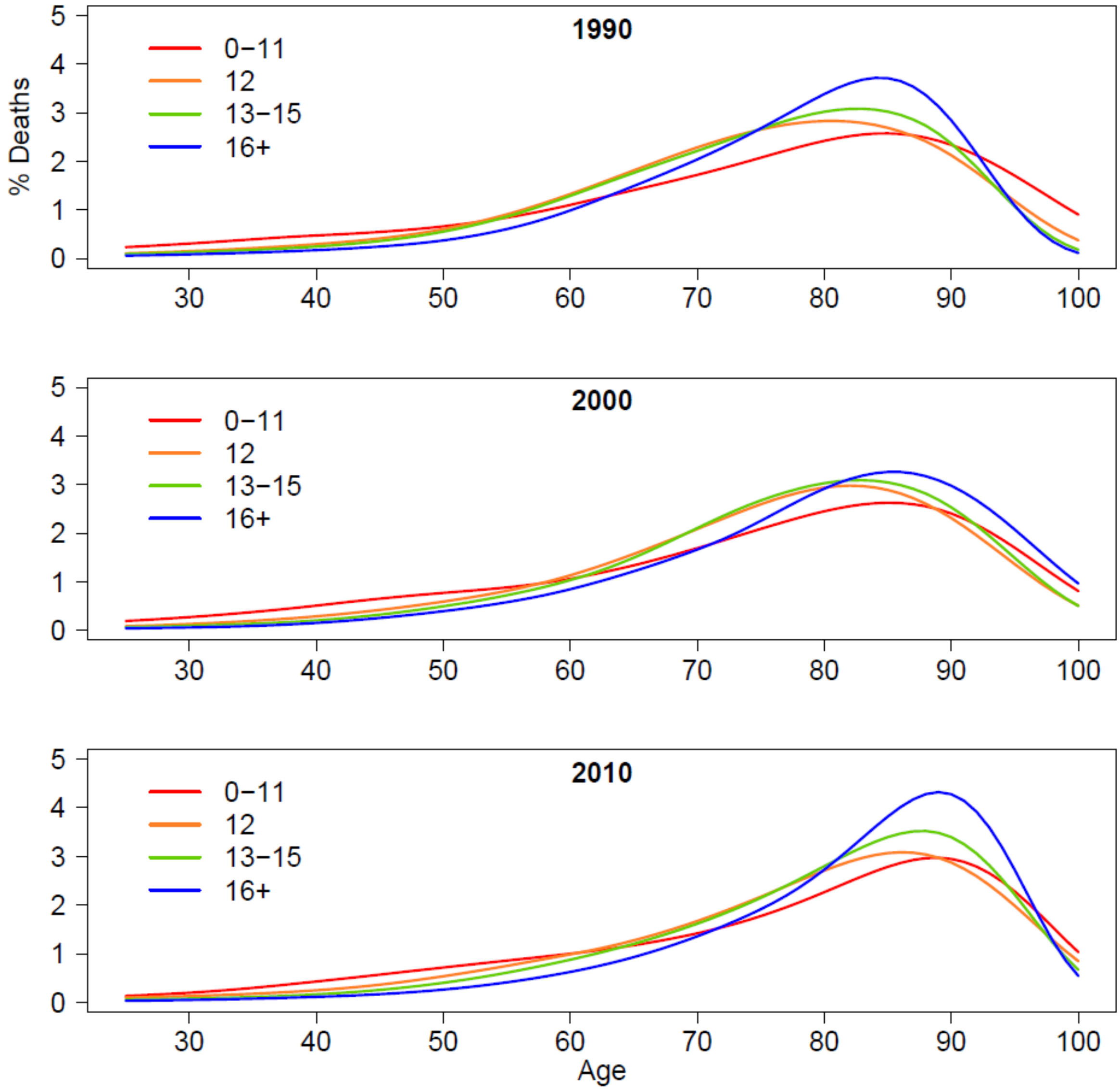

Supplement: Supplementary file 1 — (DOCX 756 kb) [file 13524_2015_453_MOESM1_ESM.pdf]
